# Countering the declining use of lithium therapy: a call to arms

**DOI:** 10.1186/s40345-023-00310-x

**Published:** 2023-08-26

**Authors:** Gin S. Malhi, Erica Bell, Maedeh Jadidi, Michael Gitlin, Michael Bauer

**Affiliations:** 1 Academic Department of Psychiatry, Kolling Institute, Northern Clinical School, Faculty of Medicine and Health, The University of Sydney, Sydney, NSW Australia; 2 CADE Clinic and Mood-T, Royal North Shore Hospital, Northern Sydney Local Health District, St. Leonards, NSW Australia; 3 Department of Psychiatry, University of Oxford, Oxford, UK; 4 Department of Psychiatry and Biobehavioral Sciences, Semel Institute for Neuroscience and Human Behavior, University of California Los Angeles (UCLA), Los Angeles, CA USA; 5 Department of Psychiatry and Psychotherapy, Faculty of Medicine, Technische Universität Dresden, Fetscherstr. 74, 01307 Dresden, Germany

## Abstract

For over half a century, it has been widely known that lithium is the most efficacious treatment for bipolar disorder. Yet, despite this, its prescription has consistently declined over this same period of time. A number of reasons for this apparent disparity between evidence and clinical practice have been proposed, including a lack of confidence amongst clinicians possibly because of an absence of training and lack of familiarity with the molecule. Simultaneously, competition has grown within the pharmacological armamentarium for bipolar disorder with newer treatments promoting an image of being safer and easier to prescribe primarily because of not necessitating plasma monitoring, which understandably is appealing to patients who then exercise their preferences accordingly. However, these somewhat incipient agents are yet to reach the standard lithium has attained in terms of its efficacy in providing prophylaxis against the seemingly inevitable recrudescence of acute episodes that punctuates the course of bipolar disorder. In addition, none of these mimics have the additional benefits of preventing suicide and perhaps providing neuroprotection. Thus, a change in strategy is urgently required, wherein myths regarding the supposed difficulties in prescribing lithium and the gravity of its side-effects are resolutely dispelled. It is this cause to which we have pledged our allegiance and it is to this end that we have penned this article.

## Introduction

Even though lithium is increasingly powering all manner of human activity, ranging from household devices to electric cars, its well-informed use in the management of bipolar disorder appears to have stagnated and is in decline, at least as measured by prescription data. This may not surprise those that regard the element as outdated, but it’s clear that its clinical utility is at odds with mounting research evidence and extensive clinical experience, not to mention clinical recommendations in the form of practice guidelines. Indeed, guidelines for the management of bipolar disorder almost all position lithium favourably, especially for long term maintenance therapy and prophylaxis (Malhi et al. 2021a, 2017; Yatham et al. 2018), and yet, in practice, it is prescribed to a diminishing number of patients with this serious mental illness that is widely acknowledged as one of the most debilitating and the most likely to result in suicide (Miller and Black 2020). Therefore, after briefly summating the statistics that reveal this declining trend, we focus in this article on the potential reasons as to why this may be the case, with a view to providing some practical suggestions as to how the prescription of lithium can be brought back in line with its genuine capabilities.

## The decline in prescriptions of lithium

As mentioned, despite lithium being endorsed widely within clinical practice guidelines, and a large body of research supporting its use in a well-defined subpopulation of patients, its use is declining. Indeed, from 1997 to 2006, the utilisation of lithium in patients diagnosed with bipolar disorder^1^ more than halved, from over 30% of patients to below 15% (Sleem and El-Mallakh 2021). A recent worldwide anonymous survey from the joined International Society for Bipolar Disorders (ISBD) / International Group for the Study of Lithium-Treated Patients (IGSLI) “Lithium Task Force” revealed that clinicians’ preferences and attitudes towards the use of lithium in the maintenance treatment of bipolar disorders appear to be affected by both the patients’ beliefs and the professional contexts where clinicians provide their services (Hidalgo-Mazzei et al. 2023). Importantly, this downward trend appears to be unique to lithium, as prescription rates for other agents utilised in the pharmacological management of bipolar disorder, such as antipsychotics and antiepileptics, have increased (Lyall et al. 2019). Furthermore, amongst mood stabilisers, lithium in particular has lower frequencies of prescription when compared to other agents. Although the decrease in lithium prescriptions is often considered to be more of an American phenomenon, the same pattern is seen in Europe (Kessing et al. 2016; Pérez de Mendiola et al. 2021). Additionally, peripartum lithium is the least prescribed mood stabiliser overall and prescription rates have dropped from 12% pre-pregnancy to as low as 2% during the second and third trimesters in one cohort study (Kan et al. 2022). Indeed, within the same study, sodium valproate, which has consistently demonstrated significant risks for congenital malformations and neurodevelopmental abnormalities (Macfarlane and Greenhalgh 2018) was prescribed in a greater proportion of women than lithium. This is despite lithium being widely regarded as safe for use during pregnancy, with studies illustrating the risk for adverse neonatal outcomes or delivery to be largely comparable to women not exposed to lithium during pregnancy or the general population (Fornaro et al. 2019). Thus, globally, the clinical use of lithium has declined consistently in the management of bipolar disorder, and even within specific populations for whom it is considered a comparatively safer choice, such as pregnant women.

## Reasons for decline in prescriptions of lithium

One of the most commonly cited reasons for not prescribing lithium is that it is widely regarded as a difficult medication to manage. This is because it is thought to require greater attention to general medical aspects of patient care than other medications. In short, it is seen as a somewhat cumbersome medication to prescribe, and at first pass there is indeed some truth to this in that the dose needs to be titrated, blood levels need to be monitored, and lithium is associated with both acute and chronic side effects some of which are potentially serious (McKnight et al. 2017). However, a distinction needs to be drawn between actual difficulties and difficulties that are perceived or have been exaggerated. For example, the need to titrate lithium is in line with good practice. We argue that it should be the case that medications when first commenced, are introduced gradually so as to avoid side effects, and ensure that only the exact amount required is being prescribed.

Similarly, the need to monitor the level of lithium in the blood, is not simply to avoid side effects, it also has the advantage of ensuring that an actual therapeutic level is achieved (Malhi et al. 2016). In fact, knowing the blood levels of a medication is of major importance, as it means that one can be certain that the medication is being taken by the patient as prescribed and that a sufficient amount is reaching the brain.

With most psychotropic medications, their blood and cerebrospinal fluid levels are assumed to be appropriate if patients take the prescribed dose as indicated. However, studies have shown that patients often do not take medications as prescribed (Jawad et al. 2018) and even if they do there is no guarantee that the medication levels needed are actually achieved within the brain. This is because of innate inter-individual differences and variability in pharmacokinetic factors such as diet, fluid intake and the effects of concurrent medications.

An additional benefit of monitoring plasma lithium levels is that it ensures engagement with clinicians and nursing staff. It also instils a practical purpose to the clinical interaction and firmly frames the management of bipolar disorder within the medical model of care. Nevertheless, concerns regarding side effects are valid, in that lithium therapy may cause a change of taste, polyuria, and thirst and in some cases result in a noticeable and troublesome tremor, and all of these add to the burden the person is already experiencing because of the illness. However, in most cases, most of these side effects can be managed satisfactorily and in fact they are often only transient and occur when initiating lithium therapy or the dose of the medication is being titrated.

Similarly, the chronic side effects of lithium, whilst very real, can be reduced by monitoring the lithium level closely and ensuring that the lowest plasma levels necessary are maintained. In addition, where possible, dosing of medication should also ensure that peak lithium levels are maintained within the therapeutic window. These precautions are likely to reduce the chances of thyroid dysfunction and long-term renal problems. Clearly, there are instances where lithium needs to be used with caution, for example during pregnancy, even though the additional risks are minimal. However, overall, it is our view that these concerns regarding lithium therapy are grossly exaggerated and that they need to be countered robustly. This can perhaps be achieved by reframing clinicians’ and patients’ worries regarding side effects and providing a more accurate picture for clinical practice.

## Lacklustre: old, not new

One of the reasons lithium is in decline is that, for many clinicians and patients alike, it cannot compete with new molecules. New medications for the management of bipolar disorder are often marketed as novel, innovative treatments, and promoted as improving upon existing therapies. In this context, lithium is decades old and consequently lacks the appeal of something new and ‘shiny’. Further, in the absence of marketing and advertising, lithium often remains out of sight and out of mind and when prescribing treatment clinicians are more likely to consider alternative, newer medications.

In clinical trials, direct comparisons are often made with lithium using statements such as “*it is superior to lithium because….*”. This not only promotes the alternative molecule, but it reinforces the potential limitations of lithium therapy. For instance, purveyors of newer agents often claim that their product does not require blood sampling and that their side effect profile is more favourable. This comparative strategy and the use of relativistic language elevates the status of alternative much newer agents and simultaneously demotes lithium further, pushing it almost completely out of contention for consideration when formulating management. We maintain that this approach is unhelpful.

Part of the reason for our concern is that this is not a valid comparison to compare the profile of a newly minted medication versus a therapy that has been established for decades. This is because the side effects of many agents only come to light after thousands of patients have trialled the treatment, and this inevitably takes several years. This is especially the case when considering the effects of chronic treatment. For example, it is now widely known that olanzapine and quetiapine have the potential to cause significant metabolic and cardiovascular side effects (Townsend et al. 2018; Weeke et al. 2014). However, these risks only became known after the molecules had been clinically available for many years and steps to avert the effects of these treatments took nearly a decade to implement.

Thus, a genuine comparison of a new agent with lithium is only possible once the treatment has been widely used, otherwise the differences between a new medication and one that is well established will necessarily favour the former and be stark because the older treatment has had more time for its side effects to come to light. In addition, other confounding factors which are not considered in clinical trials, such as medical comorbidities that are common in the patient population the drug is targeting, may also contribute to its risk profile. For example, patients with bipolar disorder have an increased risk of obesity (Kambey et al. 2023) which may augment the metabolic impact of medications (Townsend et al. 2018).

Clearly, promotional strategies of new medications are designed to capture as large a proportion of the market as possible and we appreciate that this is an essential goal for any company that has developed a new therapeutic agent. However, a more beneficial strategy would be to firstly aim to develop new treatments that complement those already in existence, and secondly ensure that they are at least on par with existing treatments in terms of efficacy and tolerability before any claims of superiority are made.

**Fig. 1:**
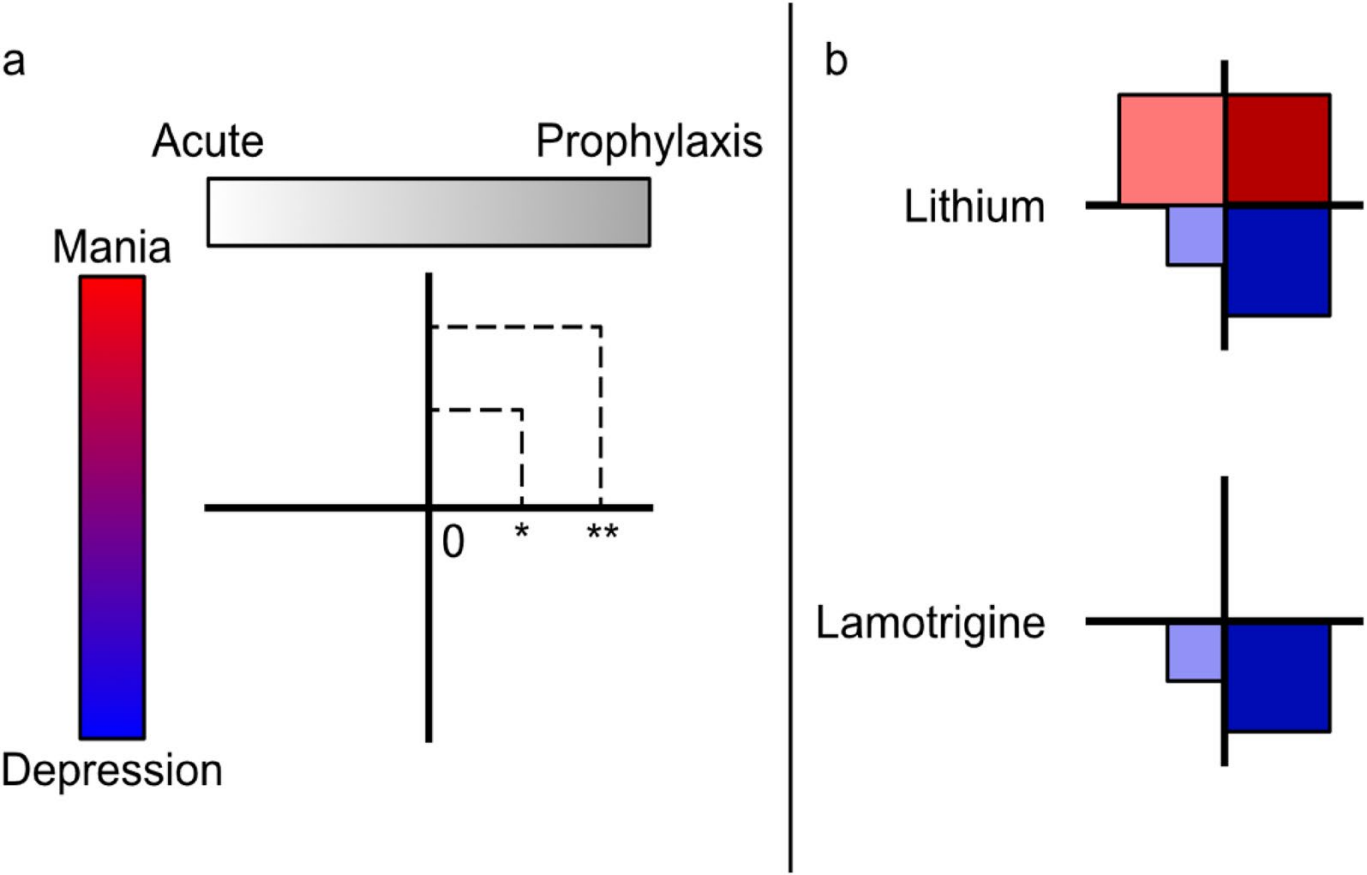
Comparison of effectiveness of mood stabilisers (adapted from Malhi et al. 2018). This schematic utilises a framework (panel a) to illustrate the clinical effectiveness of mood stabilisers, according to their strengths in managing depressive and manic episodes, both acutely and prophylactically. Lithium is effective in treating both manic and depressive episodes, although for depressive episodes in particular, it is most effective when utilised prophylactically rather than acutely. Lamotrigine is only effective in managing depressive episodes, and here again it is best utilised prophylactically rather than acutely. (* = modest efficacy; ** = significant efficacy).

In this context, it is useful to briefly consider lamotrigine, which serves as a good example of a medication that has secured a role in the management of bipolar disorder alongside lithium rather than necessarily as an alternative. This is partly because it has a beneficial effect on the depressive phase of bipolar disorder, which is not lithium's strongest suit and at the same time, it is well tolerated long term. However, it must be borne in mind that lamotrigine is not an anti-manic agent per se and given that this is a critical component of bipolar disorder, its clinical profile is necessarily very different to that of lithium (see Fig. 1, adapted from Malhi et al. 2018) (Pérez de Mendiola et al. 2021).

## Clinical profile: lack of awareness and lack of application

Alongside the decline in the prescription of lithium for the treatment of bipolar disorder, when on occasion it is considered as a therapeutic option, it is the potential side effects of lithium therapy that usually take centre stage. This is because its benefits, and in particular in whom these are most likely to be evidenced, have been largely forgotten. Indeed, there is diminishing awareness of the clinical profile for which lithium is known to be most effective. This is concerning and perhaps another reason why the use of lithium is falling. It is important therefore to emphasise that amongst the pharmacotherapeutic armamentarium available to treat both the acute episodes of bipolar disorder and maintain mood stability, lithium undoubtedly has the best-characterised clinical phenotype. This is important because it means the rate of success in patients that have a favourable clinical profile is far higher. At the same time, it means that in the absence of a favourable clinical profile, lithium should perhaps be prescribed with some circumspection.

**Fig. 2:**
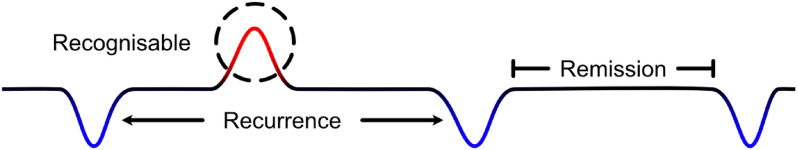
Clinical profile of lithium responders (adapted from Gershon et al. 2009). Lithium responders have clear-cut mood episodes (Recognisable), an episodic pattern of illness (Recurrence), and have periods that are symptom-free (Remission)

The clinical profile of a lithium responder has been characterised from an admixture of research studies and clinical experience. It includes patient characteristics and a distinctive pattern of illness, which has been summarised by Gershon et al. (2009). The three key features that define the clinical pattern of bipolar disorder that best responds to lithium is one in which there are well demarcated episodes of acute illness (depression and mania). As such they are *r*ecognizable. These acute episodes of illness are *r*ecurrent and are separated by equally discernible periods of *r*emission. This pattern can be remembered as the three R’s (**r**ecognizable episodes, that are **r**ecurrent and separated by periods of **r**emission) (See Fig. 2). In addition, clinical factors such as family history of a similar pattern and good response to lithium add to the probability that the individual will respond favourably to lithium. In practice, a third of patients with bipolar disorder can be expected to respond well to lithium therapy (Grof 1999). Puzzlingly, this knowledge seems to be petering out in clinical practice and has either been forgotten or is not being applied. At the same time, the clinical profile of patients and the pattern of their illness is not a major consideration when prescribing treatment. This is because patients with bipolar disorder are seldom profiled in this manner and instead increasing importance is being attached to disorder subtypes and patient preference. Further, when considering various therapeutic options, the question of which medication is best suited to treat this patient based on their clinical profile and pattern of illness is seldom asked. Instead, the choice of medication is based on efficacy and tolerability alone—independent of clinical considerations.

The fact that knowledge regarding a lithium responder is becoming clinically obsolete and that the treatment of bipolar disorder is planned without profiling patients and their pattern of illness is a major concern. Instead, attention is focused on subtyping bipolar disorder—even though this does not reliably inform therapeutic choice (Gitlin and Malhi 2020; Angst et al. 2019). Ironically, subtyping does not extend to the one area where it would be useful namely, identifying those patients with mixed mood states. These are remarkably common with about a third of patients experiencing symptoms of both depression and mania concurrently (Judd and Akiskal 2003).

Thus, a more logical approach would be to consider lithium first, not simply first line (See Malhi and Bauer 2023) and prescribe lithium in those that have a lithium responsive clinical profile. Those that do not respond may still be prescribed lithium to see if it confers some benefit but at the same time, alternatives may also be considered. Then, in those patients who do not respond to lithium, alternatives can be trialled while also re-evaluating the diagnosis.

## A strategic change in strategy

Noting the problems we have discussed, and the fact that despite increased knowledge and clear demonstrable superiority of lithium over other agents, its use is declining it is evident that alternative strategies to those that are currently employed are urgently needed to secure its use into the future.

An initial step that would be immediately impactful would be to affirm the truth about lithium and dispel the many myths surrounding its use (Malhi et al. 2021b). However, simply raising awareness seems to be ineffective and a more targeted approach is needed that addresses specifically the concerns that patients and clinicians maintain. An example would be reframing lithium monitoring as beneficial and a unique feature of lithium that improves patient care and enhances engagement. In other words, it is in fact a positive aspect of therapy and not a drawback. Furthermore, implementing education how to convey positive aspects and information to the patient should be an important part of educational interventions aimed to increase the use of lithium and should target clinicians early in their training—using ideally active learning methods, such as hands-on workshops, games and role-playing, which may help prescribers deal with patients that reject the use of lithium (Gomes et al. 2022).

At the same time, it has to be made clear that lithium is *not* for everyone but that a significant proportion of patients with bipolar disorder (approximately a third) will respond well to lithium therapy. Instantiating a realistic and honest approach is likely to gain greater traction with both patients and clinicians. At the same time, the myths concerning its use particularly around side effects and the inconvenience that is perceived rather than actual, need to be actively dispelled and displaced. This requires a paradigm shift in management and almost a forced insertion of lithium into management considerations. For instance, just as asking about suicidal ideation and suicidal risk have become a mantra, perhaps the adoption of a clinical slogan such as ‘lithium first’, may be useful whenever contemplating the management of a patient with bipolar disorder (Malhi and Bauer 2023).

## Data Availability

Not applicable.

## Footnotes

1 The cited study examined bipolar disorder type I specifically.
